# CAPE and Neuroprotection: A Review

**DOI:** 10.3390/biom11020176

**Published:** 2021-01-28

**Authors:** Marwa Balaha, Barbara De Filippis, Amelia Cataldi, Viviana di Giacomo

**Affiliations:** 1 Department of Pharmacy, University “G. d’Annunzio”, Chieti-Pescara, 66100 Chieti, Italy; marwa.balaha@unich.it (M.B.); barbara.defilippis@unich.it (B.D.F.); viviana.digiacomo@unich.it (V.d.G.); 2 Department of Pharmaceutical Chemistry, Faculty of Pharmacy, Kafrelsheikh University, 33516 Kafr El Sheikh, Egypt

**Keywords:** caffeic acid phenethyl ester (CAPE), neuroprotection, neurodegenerative disease, propolis, CNS injury, CNS ischemia, CNS cancer, neuroinflammation, CAPE derivatives, neurotoxic substance(s)

## Abstract

Propolis, a product of the honey bee, has been used in traditional medicine for many years. A hydrophobic bioactive polyphenolic ester, caffeic acid phenethyl ester (CAPE), is one of the most extensively investigated active components of propolis. Several studies have indicated that CAPE has a broad spectrum of pharmacological activities as anti-oxidant, anti-inflammatory, anti-viral, anti-fungal, anti-proliferative, and anti-neoplastic properties. This review largely describes CAPE neuroprotective effects in many different conditions and summarizes its molecular mechanisms of action. CAPE was found to have a neuroprotective effect on different neurodegenerative disorders. At the basis of these effects, CAPE has the ability to protect neurons from several underlying causes of various human neurologic diseases, such as oxidative stress, apoptosis dysregulation, and brain inflammation. CAPE can also protect the nervous system from some diseases which negatively affect it, such as diabetes, septic shock, and hepatic encephalopathy, while numerous studies have demonstrated the neuroprotective effects of CAPE against adverse reactions induced by different neurotoxic substances. The potential role of CAPE in protecting the central nervous system (CNS) from secondary injury following various CNS ischemic conditions and CAPE anti-cancer activity in CNS is also reviewed. The structure–activity relationship of CAPE synthetic derivatives is discussed as well.

## 1. Introduction

Propolis, a honeybee product, is a resinous product obtained from different plant parts mixed with beeswax and bee salivary enzymes and represents a multimillion dollar market [1,2]. It has been used in traditional medicine for many years due to its anti-microbial, anti-inflammatory, anti-oxidant, immunomodulator, anti-mutagenic, and carcinostatic effects [3]. Propolis consists of a large number of different compounds according to the ingredients the bees collect from the plants. Its main components are phenols and related compounds. Investigations have been conducted to purify and determine which substances are responsible for these effects. The results of these investigations have suggested that caffeic acid phenethyl ester (CAPE, phenethyl 3-(3-4 dihydroxyphenyl) acrylate) is the main component that mediates most of the beneficial effects ascribed to propolis [4]. As a consequence, CAPE has become one of the most studied natural product research topics in recent years. Grunberger and co-workers extracted and described CAPE in 1988 [5], and later Sigma-Aldrich produced commercial preparations for the market [6]. CAPE is a polyphenol with hydroxyl groups within the catechol ring which provide strong antioxidant properties to the molecule and can affect many biological activities. It has a lipophilic property because of its long carbon groups in an aromatic and aliphatic structure, which leads to adequate blood concentration after intraperitoneal administration [7]. Celli et al. [8] investigated the stability of CAPE in rats and human plasma. It was found that CAPE undergoes hydrolysis to caffeic acid after 6 h in rat plasma in vitro and also in vivo, giving caffeic acid as the major metabolite. This type of hydrolysis does not occur in human plasma because it does not contain carboxylesterase, which is responsible for CAPE hydrolysis [9]. The broad spectrum of pharmacological activities shown by CAPE ranges from anti-oxidant/anti-inflammatory to anti-viral/anti-fungal properties, including anti-proliferative effects in various cancer models [10]. A recent in vivo study indicated that CAPE has the ability to cross the blood–brain barrier in rats [11,12]. This review focuses on the protective effects of CAPE in many diseases that can affect the central nervous system (CNS). CAPE was found to have a protective effect on different neurodegenerative disorders, either those occurring with age, such as Alzheimer’s disease (AD) and Parkinson’s disease (PD), or other neurodegenerative disorders, such as amyotrophic lateral sclerosis (ALS) and seizures. CAPE was proven to protect neurons from the main underlying causes of several human neurologic diseases—namely, oxidative stress, apoptosis dysregulation, and brain inflammation. In addition, CAPE was found to be able to protect the nervous system from the consequences of some diseases not directly affecting it, such as diabetes, septic shock, and hepatic encephalopathy (HE). A paragraph about the neuroprotective effects of CAPE against adverse reactions induced by different neurotoxic substances such as ethanol, methotrexate, acrolein, and others is also provided. The role of CAPE in the pathophysiology of CNS tumors and in neuronal injuries is carefully reviewed through the description of the modulatory activity of CAPE against biochemical and histopathological cascade mechanisms following ischemic conditions in the brain and spinal cord. Finally, a brief overview of CAPE synthetic derivatives follows. Five tables summarize the main findings on CAPE effects in different areas of neuroprotection. In the column named “Parameters measured”, the effect of CAPE is reported between parentheses where applicable. In some case, the effect of CAPE is not directly deducible, but depends on the experimental model or conditions. This review provides a useful insight into the role of CAPE in the management of numerous pathological conditions affecting the CNS. The description of the molecular mechanisms and the focus on CAPE derivatives can help in discovering new promising targets for neuroprotection and in finely tailoring structural modifications of CAPE derivatives.

## 2. Methodology

The online database PubMed was used for screening the studies for this review, with no chronological limits applied. Keywords used to search for articles included caffeic acid phenylethyl ester and neuroprotection, neurodegenerative disorders, CNS injury, CNS ischemia, CNS cancer, neuroinflammation, brain injury, spinal cord injury, and neuronal injury. The search retrieved more than 90 results, including three reviews with a section or hints about neuroprotective effect of CAPE or its protective effects against ischemia-reperfusion injury. None of these discussed the diverse signaling pathways involved. After having removed duplicates and narrowing the results according to the relevance to the core topic of the review, 77 papers were selected. A thorough analysis of the reference list of all the included studies identified additional relevant studies which, together with papers cited to introduce and clarify the various subjects, led to a total of 90 references.

## 3. CAPE Effects on Different Neurologic Disorders

With age, a lot of neurologic disorders become common, such as Alzheimer’s disease (AD), Parkinson’s disease (PD), and amyotrophic lateral sclerosis (ALS). Common causes for the development of these diseases are oxidative stress, inflammation, and apoptosis impairment. A main goal of anti-ageing strategies is the elimination of harmful agents—in other words, free radicals—from the environment to protect organs from aging and other oxidative stress-related pathologies. One study evaluated the effects of the long-term administration of CAPE on histological and biochemical alterations induced by ageing in the brain and cerebellum of old rats. CAPE increased the superoxide dismutase (SOD), catalase (CAT), and glutathione peroxidase (GSH-Px) activities and glutathione (GSH) levels in both cerebral and cerebellar tissues to levels similar to those in young rats. Additionally, CAPE reduced the malondialdehyde (MDA, an important marker of lipid peroxidation) level and ageing-induced ultrastructural alterations, proving to be more effective than melatonin, which is used worldwide in order to prevent ageing-related pathologies [13]. Many studies addressing the protective effects of CAPE on different neurologic disorders are described below and summarized in Table 1. Other than the above-mentioned pathological conditions, the role of CAPE in psychosis, seizures, and the side-effects of other diseases are described.

### 3.1. Apoptosis

Apoptosis dysregulation has been suggested to be the underlying cause of several human neurologic diseases. Transient focal or global cerebral ischemia in rodents leads to neuronal apoptosis. Reactive oxygen species (ROS) production is thought to represent a relevant mechanism in the series of biochemical events ultimately leading to apoptosis. CAPE blocks neuronal death through inhibition of inflammation and of mitochondrial cytochrome c release, and through a reduction in free radical generation [14,15]. Primary cultures of cerebellar granule neurons (CGNs) are considered a suitable in vitro model to study the mechanisms of neuronal apoptosis, which can be induced by low K^+^ concentrations. CAPE exerts its anti-apoptotic effect on CGNs by blocking ROS formation and inhibiting the activity of both caspase-3 and caspase-9. Caspase-3 is the executioner caspase that is downstream of the intrinsic (mitochondrial) caspase-9 and extrinsic receptor activated (Fas) caspase-8 pathways. Interestingly, CAPE was found to completely block the activation of nuclear factor-κB (NF-κB) without interfering with the marked decrease in intracellular Ca^2+^ concentration induced by low K^+^ [14,16].

Another study investigated CAPE’s neuroprotective effect against apoptotic cell death in the developing rat brain after pentylenetetrazole (PTZ)-induced status epilepticus (SE). Prolonged seizures are associated with inadequate blood flow, increased excitatory amino acid release, and decreased glucose use and oxygen consumption. All these features are associated with impaired mitochondrial function and irreversible neuronal damage. CAPE showed diminished apoptosis and preservation of the number of neurons, as well as a reduced number of caspase-3-positive cells in the hippocampus and in the prefrontal cortex [17]. Another study [18] investigated the effect of CAPE on CGNs exposed to excitotoxicity by the overstimulation of glutamate receptors. Neuronal death caused by excitotoxicity plays an important role in a number of neurodegenerative disorders, including Alzheimer’s disease, Parkinson’s disease, and multiple sclerosis. CAPE inhibited necrosis mediated by p38 phosphorylation and apoptosis mediated by cytochrome *c* release and caspase-3 activation, while it appeared to have no effect on glutamate-induced neuronal death via the NF-κB pathway. Interestingly, CAPE proved effective in modulating neurotogenesis—i.e., the formation of new neurites—in PC12 cells challenged with the dopaminergic neurotoxin 1-methyl-4-phenylpyridinium (MPP+). CAPE induced the formation, elongation, and ramification of neurites and inhibited the shortage of neurites induced by the neurotoxin. These effects were associated with an increased expression of neuronal typical proteins responsible for axonal growth (GAP-43) and synaptogenesis (synaptophysin and synapsin I) [19].

**Table 1:** CAPE protective effects in different neurological disorders. Where applicable, the effect of CAPE on the parameters measured is reported between parentheses (+, − or =).

Stimulus	Parameters Measured	CAPE Dose(s)	Experimental Model	Ref.
Aging: 18 month old rats	-Histopathological assessment -MDA (−) -SOD (+) -CAT (+) -GSH-Px (+) -GSH (+)	15 mg/kg/day, i.p., for 95 days	Male Sprague Dawley rats	[13]
Apoptosis: serum-free medium with low K^+^ (5 mM KCl)	-Apoptosis (−) -ROS (−) -Ca^2+^ (=) -NF-κB (−) -Caspase-3 and -9 (−)	10 µg/mL	Primary CGNs from 8-day-old Wistar rats	[14]
SE: PTZ 40 mg/kg followed by 10 mg/kg every 10 min until SE occurrence, i.p.	-Histopathological assessment -Caspase-3 (−)	30 mg/kg /day, i.p., for 5 days starting 40 min after the SE tonic phase	Dams reared Wistar male rats	[17]
Excitotoxicity: -Cells: glutamate 30 µM/24 h -Isolated mitochondria: glutamate and maleate (both 5 mM)	-Cell viability (+) -Caspase-3 (−) -Cytochrome c (−) -Glutamate-evoked currents	0 µM–200 µM, pre- and co-treatment	-CGNs from 8-day-old Sprague Dawley rats -Mitochondria from CGNs and livers	[18]
Cytotoxicity: MPP^+^ 100, 500 or 1000 µM	-Cell differentiation (+) -Cell viability (+) -Protein content (+) -Synaptophysin (+) -GAP-43 (+) -Synapsin I (+)	1, 5 or 10 µM	PC-12 cells	[19]
Neuroinflammation: IFN-γ and LPS	-NF-κB (−) -TNF-α (−) -NOS-2 (iNOS) (−) -CREB (+)	4 to 100 µM, 30 min before and during LPS exposure	Organotypic hippocampal cultures from the hippocampi of 5–7- day-old Wistar rats	[20]
Neuroinflammation: TNF-α 10 ng/mL/6 h	-CCL-2 (−) -CXCL-8 (−) -ICAM-1 (−) -Monocyte Adhesion (-) -DNA-binding activity of NF-κB and AP-1 (−) -IκBα -IKK -TRAF2 -TAK1 -MKK4 -JNKs -c-Jun	30 µM, pretreatment	-CRT-MG human astroglial cells -U937 human monocytic cells	[21]
XALD: human skin fibroblasts derived from XALD (GM04932, GM04934), and AMN (GM07531) patients	-TNF-α (−) -ROS (−) -NO (−) -Fatty acids (−)	1–5 µM	-Fibroblasts -Mouse primary mixed glia and astrocytes	[22]
EAE: 50 µg of guinea pig MBP and 7 mg/mL heat-killed Mycobacterium tuberculosis, intradermally.	-Neurological assessment -MDA (−) -NO (−) -XO -GSH-Px -ADA -SOD	25 µmol/kg/day, i.p., for 14 days after immunization	Female Wistar rats	[23]
ALS: transfection with pIRESneo and/or SOD1 mutants	-In silico analysis -DCF -Cell viability (+) -Nrf2 (+) -5- LO -NF-κB (−)	10 µM, co-treatment	NSC34 mouse motor neurons	[24]
ALS: SOD1G93A mutated mice	-CAPE level -Behavioural assessment - pp38 (−)	10 mg/kg/day, orally, for 7 days after disease onset	SOD1^G93A^ mice	[25]
-Neuroinflammation, in vitro: 200 ng/mL LPS -in vivo, a single intraperitoneal injection of 20 mg/kg LPS, i.p., 2 h after the last CAPE injection	-Cell viability -ERK2 -Akt -p38, -pERK1/2 -pp38 -pAKT -pJNK -EPO (+) -HO-1 (+) -iNOS (−) -COX-2 (−) -pAMPKα (+)	-0.1 to 1.75 µM 30 min before LPS treatment, or co-treatment -1 or 5 mg/kg once daily for 3 days	-BV-2 murine microglial cell line -Eight-week-old male ICR mice	[26]
PD: 6-OHDA 70 µM for 6 h, on day 8–10	-Cell viability (+) -Cytochrome c (−) -Caspase-3 (−) -Ca^2+^	10 to 100 µM, pre-treatment for 4 h	-Primary CGNs from 8-day-old Wistar rats -Rat liver mitochondria from 7-day-old Sprague–Dawley rats	[27]
Dopaminergic neurodegeneration: 6-OHDA, 40 μM for RMN and 70 μM for CGN	-Free radicals (−) -Peroxynitrite (−)	10 μM, pre-treatment for 2 h	-Rat RMN -Primary CGNs	[28]
PD: 6-OHDA 8 mg/mL, s.i.	-Fe, Cu, Zn and Mn (−) -ROS (−) -Protein content -TH -Mitochondrial functions: Ca^2+^-induced swelling, Ca^2+^ uptake and respiration	-In Vivo: 10 μmol/kg/day, i.p., 5 days -In Vitro: 0.5 or 10 µM	Wistar rats	[29]
-Dopaminergic neurodegeneration, in vitro: LPS/72 h -PD, in vivo: LPS 3 µg/µL, intranigral, or 6-hydroxydopamine 2 µg/µL, intrastriatal, 30 min after first CAPE injection.	-NO (−) -ERK -p38 MAPK -HO-1 (+) -BDNF (+) -Nrf2	-In Vitro: 3–30 µM -In Vivo: 10 or 30 mg/kg/day, i.p., for 4 days	-In Vitro: rat organotypic midbrain slice cultures -In Vivo: mouse model of dopaminergic neurodegeneration	[30]
PD: rotenone1 mg/kg, s.c., every 48 h, 9 injections	-Behavioural assessment -Histopathological assessment -CD11b -COX-2 (−) -iNOS (−) -NF-κB (−) -Dopamine level (+) -TNF-α (−) -IL-1β	2.5, 5 or 10 mg/kg/day. orally, every 48 h, 9 doses	Male Swiss albino mice	[31]
PD: CPF 80 mg/kg, s.c.	-PON1 activity (+) -Lipid profile -TSA (+) -TAC (+) -TOC (−) -Histopathological assessment	10 μmol/kg/day, i.p., 21 days	Male Swiss albino mice	[32]
PD: MPTP–HCl 20 mg/kg, i.p., four in at 2 h intervals	-TH-positive neurons (+) -Cell viability (+) -CAPE and MPP+ levels -DA (+) -MAO (−) -i- and nNOS (−) -Caspase-1 (−) -Cytochrome c (−) -AIF (−) -Free radicals (−) -Peroxynitrite (−)	2, 5, or 10 mg/kg/day, 7days	Eight-week-old male C57BL/6 mice	[33]
Loss of memory (AD): STZ 3 mg/kg, bilaterally on day 1 and 3	-TBARS (−) -GSH (+) -SOD (+) -CAT (+) -Nitrite (−) -AChE (−) -TNF-α (−) -eNOS (+) -NF-κB (−) -Behavioural assessment -Histopathological assessment	6 mg/kg/day, i.p., 28 days	Wistar rats	[34]
Dementia (AD type): STZ; 3 mg/kg, on day 1 and 3, ICV	-MDA (−) -GSH (+) -TNF-α (−) -Behavioural tests	3, 6 mg/kg/day, i.p., 28 days	Wistar rats	[35]
Dementia (AD type): Aβ1-42O, unilateral stereotaxic, ICV	-Behavioural assessment -ROS (−) -Nrf2 (+) -GSH -pGSK3α/β -Caspase-9	10 mg/kg/day, i.p., 1 h after brain lesion, 10 days	Male C57Bl/6 mice	[36]
Seizures: 60 mg/kg PTZ, i.p., single dose	-Neurological assessment -MDA (−) -NO (−) -XO -SOD (+)	100 µmol/kg, i.p., 2 days prior to PTZ injection	Female Swiss albino mice	[15]
Psychosis: dizocilpine maleate (MK-801), 0.5 mg/kg/day for 5 days, i.p.	-Behavioural assessment -Histopathological assessment -MDA (−) -PC (−) -NO (−) -SOD -GSH-Px (−) -XO (−) -ADA (−) -CAT (=)	10 μmol/kg, 6 days, started one day before MK-801, i.p.	Wistar rats	[37]
Diabetes: STZ 45 mg/kg, i.p., single dose	-NO (−) -SOD -GSH-Px (−) -GSH -XO (−) -CAT (−) -MDA (−) -iNOS (−) -TNF-α (−) -IFN-γ (−) -IL-10	25 µM/kg/day, two days after STZ treatment for 60 days	Male Wistar rats	[38]
Endotoxic shock: LPS, 20 mg/kg, i.p,	-TNF-α (−) -IL-1α, -1β, -6 (−) -IL-4, -10 (+) -sICAM-1 (−) -Histopathological assessment	10 μmol/kg/day, 14 days before shock induction and a single dose 30 min after induction	Male Wister rats	[39]
Hepatic encephalopathy: thioacetamide:600 mg/kg, i.p., two doses (0 and 24 h)	-Behavioural and motor assessment -Blood ammonia (=) -ALT (−) -AST (−)	10 µmol/kg/day, i.p., starting 1 day before the first dose of thioacetamide	Male Wistar rats	[40]
Optic nerve crushing, 10 s	-Apoptosis (−) -Astrocyte migration -Cell viability (+) -NF-κB (−) -IL-6 and -8 (−) -iNOS (−) -COX-2 (−) -TNF-α (−) -CCL-2 (−)	10 μmol/kg, i.p., 10 min after the surgery	Male Sprague Dawley rats	[41]

### 3.2. Neuro-Inflammation

Brain inflammatory events are observed in several neuropathologies or in response to infectious diseases. CAPE’s ability to control neuro-inflammation was compared with that of other drugs, such as acetyl-salicylate (anti-inflammatory), dexamethasone (glucocorticoid), pyrrolidine dithiocarbamate (anti-oxidant), and SN 50 peptide (selective permeant NF-κB inhibitor) in organotypic hippocampal slice cultures. Interestingly, CAPE presented a longer-lasting control (over 48 h after a single administration) of neuro-inflammation compared to the other compounds. This effect can be ascribed to the interference CAPE exerts on several neuroinflammatory effectors, such as NF-κB, tumor necrosis factor (TNF)-α, and nitric oxide (NO). At the dose of maximal effect (100 µM), an increase in cAMP-responsive element binding protein (CREB) activity, a molecule with anti-inflammatory activity, was also observed [20]. Another study examining the anti-inflammatory effect of CAPE on activated astroglial cells found that the modulation of NF-κB activity in astroglial cells can be a potential target for the treatment of inflammatory and degenerative CNS diseases. CAPE pre-treatment abrogated the TNF-α induced expression of chemokine (C-C motif) ligand 2 (CCL-2) and intercellular adhesion molecule-1 (ICAM-1) via the inhibition of NF-κB activation in a cell-specific manner. CAPE inhibited the downstream pathways of inhibitor κB (IκB) degradation in monocytic cells while suppressing the activation of upstream IkB kinase in astroglial cells [21].

X-linked adrenoleukodystrophy (X-ALD) is a neuro-inflammatory disease associated with the demyelination of the cerebral white matter, characterized by the accumulation of very long chain fatty acids (VLCFA) caused by peroxisomal disorder due to mutations in the *ABCD1* gene. CAPE treatment corrected both the metabolic VLCFA accumulation and the secondary inflammation. The effect of CAPE was mediated by the up-regulation of Abcd2 expression and of peroxisomal β-oxidation, leading to decreased VLCFA levels in *ABCD1*-deficient U87 cells. CAPE administration reduced the expression of inducible nitric oxide synthase (iNOS), inflammatory cytokines, and the activation of NF-κB in primary astrocytes derived by *ABCD1*/*ABCD2*-silenced mice [22]. Inflammation is a major component in the pathogenesis of another disease, experimental autoimmune encephalomyelitis (EAE), which is considered the animal model of multiple sclerosis (MS). In both EAE and MS, ROS can damage the myelin sheath and the blood–brain barrier (BBB). CAPE maintains cell membrane integrity and function, thus preventing protein leakage and accumulation by inhibiting the peroxidation of membrane lipids. CAPE may exert its anti-inflammatory effect and ameliorate clinical symptoms by inhibiting ROS production at the transcriptional level, through the suppression of NF-κB activation, and by directly inhibiting the iNOS catalytic activity [23].

ALS is a disease that causes the death of motor neurons controlling voluntary muscles. In one study in 2009 [24], CAPE was selected from a library of 2000 small anti-oxidant molecules for the mechanism of action and the ability to be in the CNS in a concentration sufficient to give a therapeutic effect. In two experimental models of ALS, represented by mouse motor neuron cells (NSC34) expressing mutant superoxide dismutase 1 (SOD1) and motor neurons isolated from cases of familial SOD1-associated ALS, CAPE inhibited NF-κB-induced inflammation. CAPE also activated the nuclear factor erythroid 2-related factor 2 (Nrf2)–ARE pathway, which is usually down-regulated in ALS. Genes up-regulated by Nrf2 include GSH-Px, glutathione reductase, heme oxygenase 1 (HO-1); enzymes involved in GSH synthesis, and NADPH-regenerating enzymes. CAPE effects were tested also in an in vivo model of mice expressing a mutant superoxide dismutase (SOD1^G93A^) linked to human ALS. CAPE increased the post-onset survival and lifespan of the mice, showing a significantly greater number of surviving motor neurons and a decreased number of activated microglia and astrocytes in the lumbar region of the spinal cord. In addition, lower levels of phosphorylated p38, a mitogen-activated protein kinase that is involved in both inflammation and neuronal death, were observed in the spinal cords of SOD1^G93A^ mice [25]. Microglial activation has been widely demonstrated to mediate inflammatory processes that are crucial in several neurodegenerative disorders. CAPE was proven to inhibit cyclooxygenase-2 (COX-2) and iNOS expression and the consequent NO production both in in vitro and in vivo models of microglia activation. Anti-neuroinflammatory responses in microglial cells were mediated by 5′-adenosine monophosphate-activated protein kinase (AMPK)α, erythropoietin (EPO), and HO-1 [26].

### 3.3. Parkinson’s Disease

PD is a neurodegenerative disorder characterized by the progressive loss of dopaminergic neurons of the substantia nigra pars compacta. 6-Hydroxydopamine (6-OHDA) is a neurotoxic synthetic organic compound used to selectively destroy dopaminergic neurons in the brain to induce PD in laboratory animals. The neurotoxicity and neuronal cell death induced by 6-OHDA are due to ROS production, cytochrome c release, and subsequent caspase-3 activation. CAPE increased the viability of cerebellar granule neurons dose dependently and markedly attenuated the 6-OHDA-induced toxicity. CAPE blocked Ca^2+^-induced cytochrome c release and caspase-3 activation, but no interaction of CAPE with caspase-3 cleavage was found [27]. These results were confirmed in the same cellular model and in rostral mesencephalic neurons (RMNs), where CAPE pretreatment increased neuronal viability from 39% to 91% in CGNs and from 33% to 60% in RMNs. CAPE is able to block 6-OHDA-induced dopaminergic neuronal death through the blockage of O^2−^ and peroxynitrite generation, suggesting its potential use as a neuroprotective drug for PD [28]. With regard to the in vivo experimental models, CAPE inhibited mitochondrial permeability transition, a process that triggers cytochrome c release and caspase-3 activation, leading to neuronal death in rats treated with 6-OHDA to induce PD. The mechanism of protection involves the scavenging of free radicals and metal chelation without mitochondrial dysfunction [29].

CAPE can protect midbrain dopaminergic neurons in vitro and in vivo also by the induction of HO-1 and brain-derived neurotrophic factor (BDNF) expression [30]. Rotenone is commonly used to induce experimental PD because of its ability to block the complex I inhibition and to activate microglia, resulting in neuropathologic and phenotypic features of PD. In a rotenone Parkinsonian mice model, CAPE treatment raised the level of striatal dopamine and lessened the inflammatory burden by suppressing microglia cells and down-regulating the gene expression of COX-2, iNOS, and NF-κB. These effects led to an improvement in locomotor activity. CAPE exposure protected against rotenone-induced histopathological abnormalities and led to an increased number of surviving neurons as well [31]. In a model for PD induced by chlorpyrifos, CAPE showed positive effects on the paraoxonase (PON1) activity, levels of lipid profile, total sialic acid (TSA), total anti-oxidant capacity (TAC), and total oxidant capacity (TOC) in plasma and brain tissue, preventing neurodegeneration [32]. In a murine model of PD induced by MPTP (1-methyl-4-phenyl-1,2,3,6-tetrahydropyridine), CAPE attenuated dopaminergic neurodegeneration and dopamine loss. This effect was associated with a marked reduction in iNOS and caspase-1 expression in vivo. In Vitro, CAPE proved able to mitigate neurotoxicity by inhibiting the mitochondrial release of cytochrome c and apoptosis inducing factor (AIF) induced by MPP^+^ [33].

### 3.4. Alzheimer’s Disease

Alzheimer’s disease (AD) is an age-associated neurodegenerative disease that can be induced in rodents by the intracerebroventricular (ICV) administration of a widely used diabetogenic drug, streptozotocin (STZ). Brain PI3-kinase activity and nitric oxide production mediated by the endothelial NOS (eNOS) are involved in the memory revival function of CAPE in STZ-ICV-administered rats. The basal NF-κB activity in the rat brain was found essential for memory functions as well. The anti-apoptotic and pro-survival functions of PI3-kinase activation regulate eNOS and NFκB activity, conferring neuroprotection and improving memory [34]. A previous study by the same group showed that the CAPE down-regulation of oxidative stress and inflammation was accompanied by the amelioration of STZ-ICV-induced dementia. A great increase in the brain GSH levels and a diminution of thiobarbituric acid reactive substances (TBARS) as well as of TNF-α content were observed in the brains of rats treated with CAPE [35]. In another study, amyloid-beta oligomers were administered to mice to induce dementia and to study the AD onset and cognitive function impairment. CAPE treatment reversed cognitive deficits and improved learning and memory abilities. This action was accompanied by an induction of Nrf2 and heme oxygenase-1 via the modulation of glycogen synthase kinase 3β in the murine hippocampus [36].

### 3.5. Seizures and Psychosis

CAPE is useful as an adjunctive treatment of seizure disorders. Seizures induced by pentylenetetrazole (PTZ) are due to the activation of glutamate receptors and the inhibition of GABA, an inhibitory neurotransmitter. Glutamate receptors’ activation enhances the ROS level, which in turn enhances glutaminergic activity, but the administration of CAPE increased the latency and decreased the duration of seizures in PTZ-treated mice. CAPE protected the brain tissue from oxidative damage because of its ability to scavenge ROS, to decrease MDA concentration, to increase the anti-oxidant SOD level, and to significantly attenuate NO generation [15].

**Figure 1:**
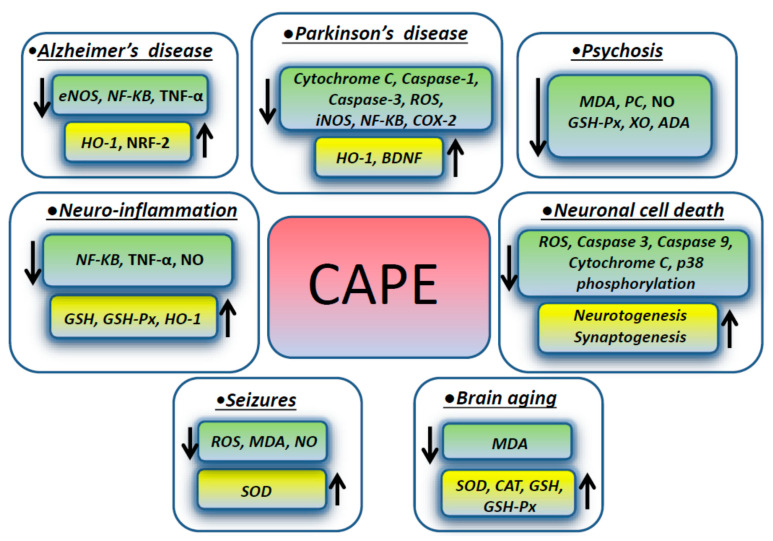
CAPE effects on intracellular molecules and pathways in different neurologic disorders.

Psychosis has many different causes and can be experimentally induced by dizocilipine maleate (MK-801), which causes neurotoxicity inducing the intracellular generation of free radical species through the N-methyl-D-aspartate (NMDA) receptor blockage. NMDA antagonists mediate cell death triggered by ROS, which are involved in membrane pathology in CNS and play a role in neuropsychiatric disorders, including schizophrenia. In one study focusing on the rat prefrontal cortex (PFC), which is the region mainly affected in schizophrenia, CAPE modulated the brain oxidant/anti-oxidant status, stabilizing the cellular membranous structures. A CAPE therapeutic effect was exerted by decreasing the levels of malondialdehyde, protein carbonyl (PC), and nitric oxide (NO). The activity of the enzymes glutathione peroxidase (GSH-Px), xanthine oxidase (XO), and adenosine deaminase (ADA) in prefrontal tissue were decreased as well, when compared to the MK-801 groups, whereas the catalase activity was not changed. In addition, CAPE treatment decreased the number of apoptotic cells in the PFC exposed to MK-801 [37]. Figure 1 summarizes the molecules involved in the effects exerted by CAPE in different neurological conditions described in the previous paragraphs.

### 3.6. Other Diseases

Some pathological conditions, different from neurodegenerative disorders, such as diabetes, septic shock, and hepatic encephalopathy (HE), can affect the health of the nervous system. Diabetes induces oxidative stress and inflammation in brain. In a murine model, CAPE significantly counteracted the effects of diabetes by decreasing the levels of nitric oxide and malondialdehyde and the activities of catalase, glutathione peroxidase, and xanthine oxidase. CAPE treatment also significantly suppressed the expression of inflammatory cytokines such as TNFα and interferon (IFN)-γ and the iNOS activity, which were remarkably enhanced in the brain by diabetes [38].

Sepsis patients suffer from severe oxidative stress, with the overproduction of reactive oxygen species and reactive nitrogen species, resulting in direct cellular injury. NF-κB plays a central role in the induction of crosstalk between cytokines and inflammatory mediators, which leads to the pathophysiology of septic shock. The effect of CAPE against lipopolysaccharide (LPS) -induced endotoxemia, neuronal damage, and the associated systemic inflammatory response was investigated in male Wistar rats. CAPE prevented neuronal damage and preserved astrocyte morphology, with no sign of inflammatory cellular infiltration, edema, or cytoplasmic swelling. CAPE decreased the levels of inflammatory cytokines (interleukin (IL)-1α, -1β, -6) and TNF-α in the plasma, increased the anti-inflammatory cytokine levels (IL-4, IL-10), and counteracted the imbalance leading to the inhibition of adhesion molecule expression (sICAM-1). This effect of CAPE on the inflammatory cellular infiltration into the brain could be directly attributed to its inhibitory effect on NF-κB activation [39].

Hepatic encephalopathy (HE) is a major neurological complication secondary to severe liver failure, causing serious neurological problems. Thioacetamide-induced HE in rats was almost fully reversed by a combination of CAPE with the laxative lactulose. The survival rates were 37.5% in the HE group, 70% in the HE + lactulose group, 80% in the HE + CAPE group, and 100% in the HE + CAPE + lactulose group. The lack of death in the animals treated with both CAPE and lactulose can be ascribed to the direct neuroprotective effect of CAPE together with the prevention of ammonia production in the body. Increased ammonia, high transaminase levels in blood, increased lipid peroxidation, and decreased antioxidant enzyme activities in most brain regions, along with impaired sensorymotor behavioral tests, were reversed to almost control values in the CAPE + lactulose-treated group [40].

Glaucoma is characterized by the death of retinal ganglion cells (RGCs) and visual field defects leading to irreversible blindness. CAPE prevented optic nerve crush-induced RGC apoptosis and neuroinflammation. These effects are mediated by the decreased expression of the inflammatory cytokines IL-8 and IL-6, inducible nitric oxide synthase, cycloooxygenase-2, tumor necrosis factor-α, and chemokine C-C ligand-2. The hypertrophy of astrocytes and Müller cells (gliosis) was modulated by CAPE through the inhibition of NF-κB signaling [41].

## 4. CAPE Protective Effects against Different Neurotoxic Substances

Some substances can affect the nervous system functions by damaging brain cells or nerves. Different studies investigating the protective effect of CAPE against different neurotoxic substances are described in this paragraph and summarized in Table 2.

The methotrexate (MTX) is widely used as a chemotherapeutic agent to treat various neoplastic diseases. However, it has a pronounced neurotoxicity which represents a relevant clinical problem, being directed towards the brain, the spinal cord, or the nerve roots. Its neuronal toxicity is primarily caused by demyelination as a consequence of axonal loss. MTX inhibits the conversion of folic acid to tetrahydrofolate, a molecule needed for the synthesis of DNA during S-phase, and leads to improper DNA synthesis and subsequent cell death. ROS production was increased indirectly by an augmented activity of purine-catabolizing enzymes such as XO and ADA. In two studies conducted in 2006 from the same group, CAPE showed protective effects in Wistar rats treated with MTX. In the first study, CAPE reversed biochemical (ADA activity and NO levels) and histopathological parameters to the control levels in spinal cord tissue [42]. In the second study, CAPE significantly reduced the MDA levels and SOD and CAT activities in rat cerebellum, leading to protection from oxidative damage caused by MTX treatment [43]. The decreased activity of the two well-known anti-oxidant enzymes SOD and CAT following CAPE administration could seem a contradiction. As a matter of fact, the latter could be explained by CAPE acting as non-enzymatic free radical scavengers, inhibiting oxidative stress with the consequent attenuation of anti-oxidant enzyme activity. The same protection from oxidative stress was found in rat spinal cord, brainstem, and sciatic nerve: CAPE treatment significantly decreased the MDA level and CAT and GSH-Px activities, whereas SOD activity was increased in comparison to the MTX group [44].

Ifosfamide (IFOS) is an alkylating chemotherapeutic agent used for a wide range of solid and hematologic malignancies. Commonly reported neurological complications of IFOS include acute alterations in consciousness, seizures, cerebral infarction, paralysis, neuropathies, leukoencephalopathy, and ototoxicity. A study in a rat model suggested that CAPE may help in counteracting IFOS toxicity and in reducing the development of changes in the brain in clinical practice. CAPE significantly decreased the MDA and PC levels, as well as caspase-3 activity, preventing neuronal apoptosis [45].

Cisplatin is another effective broad-spectrum chemotherapeutic drug. Peripheral sensory neuropathy is its major dose-limiting side effect. Recently, two studies by the same group demonstrated the protective activity of CAPE against neurotoxicity induced by cisplatin [46,47]. The first study used PC12 cells, a model of sympathetic neurons responsive to nerve growth factor (NGF). The study showed that CAPE reduced the axonal damage induced by cisplatin by promoting neuroplasticity through the activation of NGF high-affinity receptors (trkA) and the up-regulation of axonal proteins related to neurite outgrowth and synaptic communication [46]. In addition, CAPE attenuated the down-regulation of cytoskeleton proteins (F-actin and β-III-tubulin) and energy-related markers (AMPK α, p-AMPK α, and SIRT1) as well. Moreover, the neuroprotective mechanism of CAPE also involves the activation of the neurotrophic signaling pathways MAPK/Erk and PI3k/Akt [47].

Doxorubicin, one of the most widely used anticancer agents, is known to be neurotoxic and to induce oxidative injury and lipid peroxidation in the brain. One study concluded that doxorubicin-induced oxidant injury can be prevented in the rat brain by CAPE treatment, which decreased the level of MDA and increased the CAT activity [48].

Cigarettes contains various toxic substances that can be considered harmful to the nervous tissue. CAPE was found able to have an anti-apoptotic role in the hippocampal formation of rabbits exposed to cigarette smoking. CAPE-treated rabbits showed a decreased MDA level and increased SOD activity compared with untreated rabbits [49]. Similarly, brain injury in alcoholic patients can be caused by the oxidative degradation of the mitochondrial genome. The combination of CAPE and intralipid treatment has a synergistic protective effect against ethanol-induced neurotoxicity: the first decreased the total oxidant status (TOS), while the intralipid treatment increased the total antioxidant status (TAS). Histopathologic results confirmed the biochemical results [50].

Acrolein is a ubiquitous pollutant existing in alcoholic beverages, water, and foods such as cheese, donuts, and coffee, and is also formed during the combustion of organic materials, including engine exhaust, wood, tobacco, and over-heated cooking oils. Acrolein-induced oxidative stress has been implicated in the pathophysiology of neurodegenerative diseases, including Alzheimer’s disease. Acrolein induces the hyperphosphorylation of microtubule-associated protein tau and promotes amyloid-β peptide (Aβ) aggregation in senile plaques. Pretreatment with CAPE significantly attenuated acrolein-induced neurotoxicity, ROS accumulation, and GSH depletion in HT22 mouse hippocampal cells. CAPE modulated the MAPK and Akt/GSK3β signaling pathways and restored the changes induced by acrolein in β-secretase (BACE-1) activity and/or in the activation of α-secretase (ADAM-10). These findings suggest that CAPE may provide a promising approach for the treatment of acrolein-related neurodegenerative diseases [51].

Hexavalent chromium [Cr(VI)], a proven toxin and carcinogen, is commonly used in industry and its improper disposal leads to increasing levels of Cr(VI) in water, soil, and air, causing environmental pollution. Cr(VI) administration produced a significant increase in the pro-inflammatory cytokines, TNF-α and IL-6, in the rat cerebrum. Cr(VI) provoked oxidative stress and inflammation, leading to activation of the JAK/STAT signaling pathway, whose dysregulation represents a key factor in neurodegenerative diseases. The neuroprotective effects displayed by CAPE could be explained by its anti-inflammatory potential. CAPE effectively decreased lipid peroxidation and NO production, and rejuvenated the altered GSH and anti-oxidant defense enzymes. In addition, it can exert a modulatory effect on the JAK2/STAT3 pathway via a SOCS3-independent mechanism. The study demonstrated that CAPE protects the brain against Cr(VI)-induced toxicity through the attenuation of oxidative stress and inflammation [52]. Cadmium (Cd), a non-biodegradable heavy metal, is another environmental pollutant. Cd could cause damages to the central and peripheral neuronal systems and can induce hippocampal damage and memory deficits. A recent study showed cognitive deficits and spatial memory impairments due to CdCl2-induced neurotoxicity in mice. CAPE significantly decreased CdCl2-induced neuronal apoptosis and the expression of Bax and cleaved caspase-3 while promoting Bcl-2 expression in mice hippocampus. CAPE also inhibited the CdCl2- initiated Aβ accumulation and activation of pro-inflammatory factors and microglia in the brain [53].

Neurotoxicity can be also caused by poisoning with organophosphate pesticides, which results in cholinergic syndrome. Seeking an alternative or supportive treatment for organophosphate insecticide poisoning, a study investigated the effect of CAPE on chloropyriphos toxicity. Although CAPE had a significant effect on the protection of neuronal degeneration by decreasing the amount of oxidative stress (TOS), the results revealed that CAPE significantly inhibits the enzyme AChE. Consequently, it is not suitable for the management of organophosphate toxicity [54] where the acetilcholinesterase activity is already compromised.

In order to decrease the risk of drug resistance, a commonly used strategy is the combination of various drugs to treat a disease. Isoniazid (INH), a first-line drug for tubercolosis, has side effects on both the central and peripheral nervous system, while optic neuropathy has been reported with the administration of ethambutol (ETM). Tuberculosis treatment can be discontinuous because of such neurotoxic side effects. Interestingly, one study demonstrated that CAPE can protect against INH- and ETM-induced neurotoxicity in the rat brain and sciatic nerve. CAPE normalized the levels of both MDA and TOS, increased by anti-tuberculosis medications, and prevented the down-regulation of SOD and PON-1 activities by scavenging the free radicals produced following INH and ETM administration. CAPE treatment reduced effectively the edema and vascular congestion caused by these medications in both the central and peripheral nervous systems [55].

Sevoflurane, a volatile anesthetic frequently used for pediatric anesthesia, was reported to promote neurodegeneration. A recent study demonstrated that CAPE effectively inhibited sevoflurane-induced neuroapoptosis in rat pups by modulating the expression and phosphorylation of apoptotic proteins, MAPKs, and the PI3K/Akt pathway. CAPE significantly reduced sevoflurane-induced apoptosis, down-regulated the expression levels of caspases and pro-apoptotic proteins (Bax and Bad), and elevated the expression levels of Bcl-2 and Bcl-xL when compared with sevoflurane treatment [56].

## 5. CAPE Protective Effects against Ischemia

Approximately 87% of all brain strokes are ischemic ones. Ischemic stroke is caused by a blockage in an artery that supplies blood to the brain. The blockage reduces the flow of blood and oxygen to the brain, leading to the damage or death of brain cells. If circulation is not restored quickly, the brain damage can be permanent. Ischemic brain damage is an evolving process which begins during the insult and extends into the recovery period after the reperfusion interval. The immediate area surrounding the infarct consists of neurons undergoing either necrosis or apoptosis.

Many studies have investigated the potential protective role of CAPE in various CNS ischemic conditions (Table 3). In one study, the effects of CAPE and alpha-tocopherol, another free radical scavenger, on ischemia–reperfusion cerebral injury were compared. CAPE proved to be better than alpha-tocopherol in reducing the cerebral level of MDA, a marker of oxidative stress [57]. CAPE was also found able to significantly reduce the total infarct volume following focal cerebral ischemia. Its effects were found to be related to its antioxidant activity and to the up-regulation of NO production [58], which has many beneficial properties in ischemia–reperfusion injury, including an increase in blood flow produced by cerebral vasodilation. Interestingly, Khan et al. showed that the protective effect of CAPE after transient focal cerebral ischemia and following reperfusion is achieved through not only anti-oxidant and anti-inflammatory mechanisms, but also via hemodynamic effects in a dose-dependent manner in both short- and long-term ischemia/reperfusion models. CAPE increased nitric oxide and glutathione levels, decreased lipid peroxidation and nitrotyrosine levels, and enhanced cerebral blood flow. A down-regulation of the inflammation by blocking of the NF-κB activity was also observed. The affected mediators included adhesion molecules (ICAM-1 and E-selectin), cytokines (TNF-α and IL-1β), and inducible nitric oxide synthase. This action was further confirmed by a reduction in the expression of ED1, a marker of activated macrophage/microglia. CAPE also inhibited apoptotic cell death by down-regulating caspase-3 and up-regulating the anti-apoptotic protein Bcl-xL [59]. These results were supported by a study on rabbits [60], in which CAPE attenuated the elevation of the plasma level of MDA, CAT, and XO caused by cerebral ischemia injury and restored the plasma level of GSH and NO. In the same in vivo model, CAPE significantly attenuated the serum S-100B level, an index for brain damage, after middle cerebral artery occlusion [61]. As for the timing of CAPE administration, Cengiz et al. [62] showed that the pre-treatment reduced neuroglia activation and structural changes (infarcted areas, pyknotic cells, vacuolization, and neuroglial cell infiltration) in the rat cerebral cortex after middle artery cerebral ischemia/reperfusion. On the other hand, the post-treatment of ischemic brain injury with CAPE significantly reduced the infarct size, IL-1α production, and expression, in a dose-dependent manner, of TNF-α, hypoxia inducing factor (HIF)-1α, monocyte chemoattractant protein (MCP)-1, and indoleamine 2,3-dioxegenase (IDO) in the cerebral cortex ipsilateral to the photothrombosis. In addition, CAPE elicited a significant increase in HO-1 expression and IL-10 production, demonstrating that its anti-inflammatory properties are related to a remarkable neuroprotective effect on ischemic brain injury [63]. Feng and collaborators reported in 2008 [64] that CAPE compensated the functional alterations in mitochondria isolated from mouse brain challenged by anoxia-reoxygenation. This effect was attributed to the inhibition of the decrease in the membranes’ fluidity and of lipoperoxidation and protein carbonylation. A blockade of the enhanced release of cardiolipin and cytochrome c was also observed. Interestingly CAPE was used at concentrations ranging from pico- to micromolar, making CAPE more efficient in quenching free radicals than its derivatives, ferulic acid and ethyl ferulate.

The effect on isolated brain mitochondria was confirmed in another paper, where CAPE directly inhibits the Ca^2+^-induced cytochrome c release. In the same study, CAPE proved able to prevent neuronal death in neonatal rats exposed to hypoxia–ischemia. This condition is associated with clinical syndromes of neurological disability, such as seizures, intellectual impairment, and cerebral palsy. CAPE can effectively protect against neuronal and tissue loss in the cortex, hippocampus, and thalamus in vivo. In addition, CAPE inhibited hypoxia-induced caspase-3 activation and the hypoxia-mediated expression of inducible nitric oxide synthase and caspase-1 in vivo. It also potently blocked nitric oxide-induced neurotoxicity in vitro [65].

In another model of stroke, CAPE was tested on subarachnoid hemorrhage (SAH) and was found able to significantly attenuate brain ischemia due to vasoconstriction after SAH; CAPE inhibited the marked narrowing in the lumens and the thickening in the walls of the basilar arteries as well. The tissue levels of MDA were also found to be decreased after CAPE administration following SAH because of ROS scavenging, lipid peroxidation prevention, and GSH and NO increases in the cerebral tissue [66]. In a similar model, CAPE was shown to reduce the hippocampal neuron loss induced by SAH, but no significant effect of CAPE on the vasospasm was found [67].

Injury to the gray matter of the spinal cord, with consequent neuronal death, has generally been considered an important element in the pathology of spinal cord ischemic injury. The latter represents the main complication in the surgical repair of thoracic and thoraco-abdominal aneurisms and remains a persistent clinical problem. The most serious complication is paraplegia. Methylprednisolone (MP) proved effective in improving neurologic function after traumatic spinal cord injury. Ilhan et al. [68] compared the effects of CAPE and MP on histopathological changes, antioxidant status, lipid peroxidation, and neurologic recovery in temporary induced spinal cord ischemia in rabbits. CAPE reduced ischemic and reperfusion damage and provided a better neurologic outcome than MP. CAPE administration resulted in an improvement in microcirculatory environment during reperfusion, preventing endothelial cell lysis provoked by proteases released by activated leukocytes.

## 6. CAPE Protective Effects against Injury

The secondary injury consists of the biochemical and histopathological cascade mechanisms in brain and spinal cord following a primary injury and leading to progressive neuronal death after trauma. Table 4 lists the studies investigating the protective effect of CAPE against these mechanisms.

A clip compression model was used in rats to simulate the spinal cord injury (SCI). In the early phase of injury, CAPE suppressed the serum levels of pro-inflammatory cytokines—i.e., TNF-α and IL-1β. At the same time, CAPE decreased hemorrhage and necrosis occurrence both in the gray and white matter, as well as in the central canal, as demonstrated by the histopathological evaluation of the healing process [69]. Another study compared the effects of CAPE with those of methylprednisolone (MP) in the prevention of neurological deficits caused by secondary spinal cord injury. CAPE has been found to be superior to MP in preventing apoptosis without showing the latter side effects—namely, gastrointestinal hemorrhage and infection [70]. The effects of intrathecal CAPE administration following SCI were investigated as an alternative approach in CNS diseases because the systemic administration of pharmacological agents does not guarantee the attainment of the maximum effective dose in the damaged area. The intrathecal CAPE administration resulted in a decrease in the tissue and serum levels of IL-6, an inflammatory cytokine that plays an essential role in secondary damage. Although no significant decrease was identified in the TNF-α level, CAPE led to a decrease in the edema and microhemorrhage that developed in association with a local inflammatory response in the damaged area. In addition, CAPE was more effective than MP in decreasing microhemorrhage [71]. Interestingly, the intratechal administration of CAPE and MP decreased also the MDA, TOA, and SOD levels, with a greater effect in the CAPE group [72]. Since CAPE and MP treatment substantially increased the TAC levels, it was concluded that the intrathecal injection of both compounds can inhibit lipid peroxidation and oxidant increase following SCI. In a model of SCI caused by hemi-transection, CAPE suppressed the expression of the mRNA of the pro-inflammatory cytokine IL-1β and of the two inflammatory enzymes iNOS and COX-2. An enhanced recovery of locomotor function and a reduction in the lesion size were also found following CAPE treatment [73].

One of the early pathological events triggered by traumatic brain injury (TBI) is the breakdown of the blood brain barrier (BBB), which leads to the infiltration of fluid and circulating cells and molecules, resulting in an exacerbation of brain damage and tissue loss. Recently, myeloperoxidase (MPO) analysis was used to identify the level of polymorphonuclear leukocyte (PMNL) infiltration after TBI. PMNLs worsen brain damage due to the release of cytotoxic mediators that interfere with BBB function. Hypochlorite acid is formed through the MPO-H_2_O_2_–Cl-system and is one of the oxidants released by activated leukocytes. CAPE administration to mice with TBI showed a significant decrease in MPO levels, thereby inhibiting the BBB inflammatory process [74]. Another study examined the potential role of CAPE in reducing some of the pathological consequences of TBI in rodent models. The post-TBI administration of CAPE reduced the BBB permeability and cortical tissue loss both in rats and mice. The improvement was associated with the preservation of the levels of the tight junction protein claudin-5. On the other hand, it failed to offer significant improvements in either motor or memory tasks [75]. The administration of a single dose of CAPE post-TBI can also have protective effects via decreasing the elevated MDA levels and restoring the activities of antioxidant enzymes (SOD and GSH-Px), with the exception of CAT. The morphology of neurons was well preserved, and the immunoreactivity of degenerating neurons after trauma was reduced in the CAPE-treated group [76].

Since TBI has a multi-mechanistic etiology, a recent study combined the endogenous nitric oxide (NO) metabolite S-nitrosoglutathione (GSNO) with CAPE to accelerate the rate and enhance the degree of recovery. GSNO is a natural component of the human body, present in the brain and other organs. It provides neuroprotection and improves neurobehavioral functions via anti-inflammatory and anti-neurodegenerative mechanisms. It acts via the S-nitrosylation of target proteins, including NF-κB, STAT3, COX-2, caspase-3, calpains, and i- and eNOS. The combination therapy improved cognitive and depressive-like behavior compared with GSNO or CAPE monotherapy. Separately, both GSNO and CAPE improved mitochondrial integrity/function and decreased oxidative damage; however, the combined therapy had greater effects on dynamin-1-like protein (Drp1) and manganese-dependent superoxide dismutase (MnSOD). Additionally, while CAPE alone activated 5′-adenosine monophosphate-activated protein kinase (AMPK), this activation was heightened in combination with GSNO. These results recommended that the combination therapy-based multi-mechanistic approach is worthy of investigation in human TBI because no adverse effect for both GSNO and CAPE is known [77].

## 7. CAPE Anti-Tumoral Effects in CNS

In 2020, according to Cancer Stat Facts, an estimated 23,890 new patients in the USA will be diagnosed with primary cancerous tumors of the brain and spinal cord. The 5-year survival rate for people with a CNS tumor is around 32.6% [78]. The most common types of brain tumors are gliomas and meningiomas. Gliomas are considered the most malignant form of brain tumors, and are ranked among the most aggressive human cancers. Choi et al. in 2007 [79] demonstrated that the anti-oxidant property of CAPE can exert a pro-apoptotic effect in Fas-mediated cell death in human malignant astrocytoma cells. Fas activation increased the intracellular ROS levels in a NADPH oxidase- and caspase-dependent manner. Choi’s results suggested that Fas-induced ROS production protects astrocytoma CRT-MG cells from caspase-dependent apoptosis. The suppression of Fas-induced ROS levels augments Fas-mediated apoptosis by enhancing the enzymatic activity of caspase-3. CAPE, by inhibiting ROS production, dramatically sensitized astrocytoma cells to the Fas-induced loss of mitochondrial transmembrane potential and subsequent cell death. Another study demonstrated that CAPE suppressed the invasion and proliferation of glioma cells by the down-regulation of phospholipase D1 (PLD1) expression at the transcriptional level. PLD is a class of enzymes functionally linked with oncogenic signals and tumorigenesis. CAPE exerted its effect via the inhibition of the binding of NF-κB to the PLD1 promoter. In addition, CAPE bound to a Cys837 residue of the protein PLD1 and inhibited its enzymatic activity. The activation of matrix metalloproteinases-2 induced by phosphatidic acid, a product of PLD activity, was also inhibited [80].

5-lipoxygenase (5-LO) is another enzyme involved in the pathophysiology of glioma. A strong 5-LO expression has been observed in glioma cells, and inhibiting this enzyme could impact glioma cell proliferation. Interestingly, CAPE (1 µM) was found to be effective in reducing 5-LO activity more efficiently than the ferulic acid ester (FAPE) in two human glioma cell lines, Hs683 and LN319. CAPE administration led to cytotoxicity, cell growth impairment, and apoptosis occurrence [81].

In an in vivo model, Bio 30, a water-miscible CAPE-rich extract of New Zealand propolis, was analyzed for its anti-tumoral properties. Bio 30 suppressed completely the growth of both neurofibromatosis type 1 (NF1) and neurofibromatosis type 2 (NF2) tumor xenografts in mice, suggesting that CAPE-rich propolis might serve as an effective, inexpensive, and safe NF therapeutic first approach. Seventy percent of human cancers and all NF tumors, but not normal cells, are highly dependent on PAK1 for their growth. PAK1 is a Rac/CDC42-dependent Ser/Thr kinase. Evidence was found that CAPE, by blocking the oncogenic PAK1 signaling pathways, is the anti-tumor ingredient of Bio 30 [82]. All the studies described above about the anti-tumor effect of CAPE in CNS are listed in Table 5.

## 8. CAPE Derivatives

**Figure 2:**
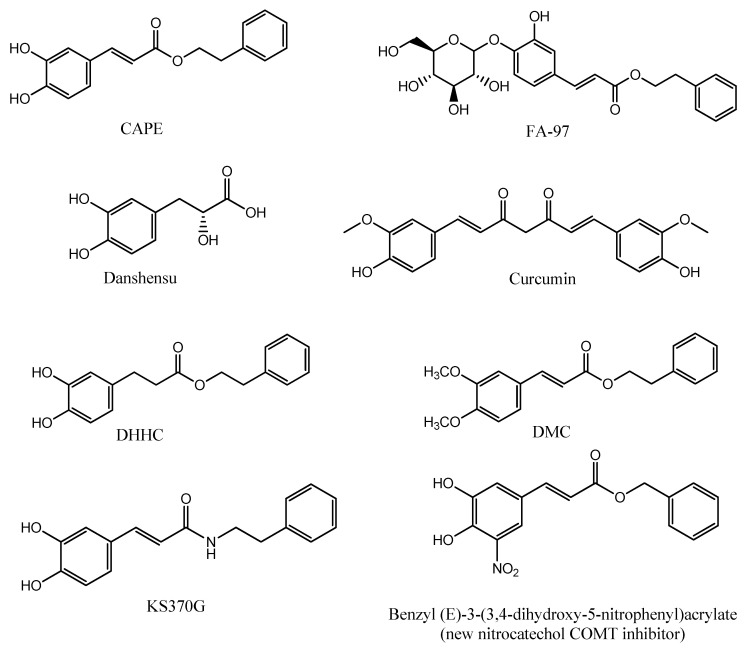
Chemical structure of CAPE and CAPE analogues.

Due to the involvement of a variety of pathways in neurological disorders, the finding of molecules with multiple neuroprotective effects would be a great achievement for the slowing down of the course of these diseases. CAPE is supposed to be a promising neuroprotective agent with multiple targets. Many studies have aimed to explore the structure–activity relationship of CAPE in order to optimize its structure through a series of studies on CAPE derivatives. Figure 2 shows the chemical structures of CAPE and some of its analogues. Novel derivatives of CAPE were recently designed, synthesized, and evaluated in different in vitro models as potential neuroprotective agents. Phenolic hydroxyl groups and double bonds in the structure of CAPE proved critical in conferring neuroprotective properties to the compound, whereas monophenol compounds showed a low anti-inflammatory activity. Compounds with a phenolic hydroxyl group on para-position exerted a higher anti-inflammatory activity than compounds with the same group on the ortho-position, indicating that conjugated double bond played an important role in anti-inflammatory activity. Only compounds with a retained catechol structure showed anti-oxidant properties. The amino group did not show the same anti-oxidant activity found for the bioisoteric and the hydroxyl group. Replacing the aromatic alkyl part of CAPE with different liposoluble groups improved the anti-inflammatory and anti-oxidant activities and the BBB permeability [83], measured with a parallel artificial membrane permeability assay (PAMPA), a high-throughput technique developed to predict passive permeability through biological membranes. These findings were supported with a study on PC12 cells suggesting that the presence of the caffeic acid group is important to maintain the cytoprotective and neuritogenic activities of the compound [84]. Another group explored the neuroprotective effects of CAPE and another two caffeic acid derivatives, Danshensu and Curcumin. They are similar in structure, since they have an aromatic ring, but they differ in the numbers of hydroxyl groups attached to the aromatic ring and in the conjugated double bond. The structure–neuroprotective activity relationship against H_2_O_2_-induced oxidative damage in PC12 cells and the D-galactose (D-gal)-induced cognitive impairment was investigated in mice. The neuroprotection exerted by these compounds was mediated by the activation of the protein kinase A (PKA)-cyclic AMP response element-binding protein (CREB) pathway. It was found that 3, 4-dihydroxyphenyl hydroxyl groups attached to the aromatic ring play a more important role than conjugated double bond in up-regulating the expression of the PKA/CREB pathway in the mouse hippocampus [85].

In two separate studies, CAPE proved active in suppressing the invasion and proliferation of glioma cells by the down-regulation of PLD1 and 5-LO expression. One study showed that CAPE analogues with a catechol moiety, dihydroxydihydrocinnamic acid phenethylester (DHHC) and dimethoxycinnamic acid phenethyl ester (DMC), inhibit PLD1 expression with a lower potency than CAPE due to differences in NF-κB transactivation. This was confirmed by structural analysis, which showed that both electrophiles in CAPE, Michael reaction acceptor and catechol moiety, are involved in the down-regulation of the PLD1 expression and in NF-κB transactivation [80]. The other study demonstrated that CAPE, but not FAPE, displayed substantial cytotoxicity against two glioma cell lines [81].

Even though CAPE stability in human plasma was demonstrated [9], its lipophilic properties, due to long carbon groups in an aromatic and aliphatic structure, can hamper CAPE absorption upon oral administration. Hence, FA-97 caffeic acid phenethyl ester 4-O-glucoside was synthesized to overcome the low water solubility and poor bioavailability of CAPE, which represent the main limits to its application in vivo. The in vivo and in vitro results showed that FA-97 is able to suppress oxidative stress-mediated neuronal cell apoptosis and to have protective properties against scopolamine-induced cognitive impairment. Since the pathway involved in such activity is the Nrf2/HO-1 signaling pathway, FA-97 can be suggested to have a therapeutic potential in AD [86]. The low bioavailability of CAPE could also be ascribed to rapid decomposition by esterases. Another study aimed to synthesize CAPE analogs with a better stability and higher BBB permeability; it was found in various in vitro models that E-3,4-dihydroxy styryl sulfonamides and their 3,4-diacetylated derivatives have better neuroprotective activities against oxidative and inflammatory injury than CAPE. The same compounds showed high blood–brain barrier permeability [87], as predicted by the PAMPA test. KS370G, a novel caffeic acid phenylethyl amide. significantly inhibited neuroinflammation in CNS by the inhibition of the NO release and of the iNOS and COX-2 expression. KS370G treatment also induced HO-1 activity and suppressed the cytokine signaling (SOCS)-3 expression in the microglia [88].

Centrally active catechol *O*-methyltransferase (COMT) inhibitors can have a role in the treatment of dopamine deficiency-related neurological disorders such as PD, depression, and schizophrenia. First-generation COMT inhibitors, such as tolcapone and entacapone, are based on simple catechol scaffolds, and their clinical utility was hampered due to hepatotoxicity in addition to poor bioavailability. Recently, new nitrocatechol COMT inhibitors (Figure 2) based on naturally occurring CAPE were developed. Their effect was within the nanomolar range with a lower hepatotoxicity in addition to a good BBB permeability [89], as evaluated by PAMPA assay. Lead optimization efforts opened a new window on repurposing of nitrocatechols beyond their established role as COMT inhibitors. The results of another study showed that the nitrocatechol scaffold is required for a significant inhibition of hyperphosphoryled tau protein aggregation. Tau hyperphosphorylation and assembly into intracellular neurofibrillary tangles triggers neurodegeneration and was largely observed in AD. The activity of these compounds was enhanced by introducing bulky substituents at the side chain and by αcyanocarboxamide derivatization, as the amide bond provides a superior conformational stability [90].

## 9. Conclusions

CAPE, the most investigated compound of propolis, has a wide range of pharmacological activities. It has a strong neuroprotective activity and its effects have been studied in several pathological conditions affecting the CNS. The main goal of this review is to provide a comprehensive view of the role of CAPE in the management of these disorders by focusing on the published data about CAPE and its neuroprotective effects. In this review, we also shed light on its molecular mechanisms of action. In addition, we summarize the structure–activity relationship of CAPE synthetic derivatives, which will be helpful for the further development of neuroprotective derivatives.

## Figures and Tables

**Table 2:** CAPE protective effects against different neurotoxic substances. Where applicable, the effect of CAPE on the parameters measured is reported between parentheses (+, − or =).

Neurotoxic Substance	Parameters Measured	CAPE Dose	Animal/Cell Used	Ref.
MTX: 20 mg/kg, i.p., single dose	-Histopathological assessment -ADA (−) -NO (−)	10 µmol/kg/day, i.p., for 7 days	Male rats	[42]
MTX: 20 mg/kg, i.p., single dose on day 2	-MDA (−) -SOD (−) -CAT (−)	10 µmol/kg/day, i.p., for 7 days	Male rats (cerebellum)	[43]
MTX: 20 mg/kg, i.p., single dose on day 2.	-MDA (−) -SOD (−) -GSH-Px (−) -CAT (−)	10 μmol/kg/day, i.p., for 7 days	Wistar male rats (spinal cord, sciatic nerve, brain stem)	[44]
IFOS: 300 and 500 mg/kg, i.p., two doses	-Carbonyl content (−) -CAT -MDA (−) -Caspase-3 (−)	10 µmol/kg/days, i.p., for 2 days, starting 1 day before injection of IFOS	Wistar male rats	[45]
Cisplatin: 5 and 32 μM	-Cell viability (+) -Neurite outgrowth (+) -GAP-43 (+) -Synapsin I (+) -Synaptophysin (+)	1, 5, 10, 25, 50, and 100 μM for 24 h	-PC12 cells -SH-SY5Y cells	[46]
Cisplatin: 5 μM	-Protein content (+) -Glucose uptake (+) -Glutamate uptake -ROS (−) -F-Actin (+) -β-III-Tubulin (+) -SIRT 1 (+) -AMPK α and pAMPK α (+)	10 μM	-PC12 cells -transfected COS-7 cells -transfected HEK cells -glial cells	[47]
Doxorubicin: 20 mg/kg i.p., single dose	-MDA (−) -NO (−) -GSH-Px -CAT (+) -SOD	10 μmol/kg/day, i.p., for 12 days starting 2 days before doxorubicin	Male Sprague Dawley rats	[48]
Cigarette smoke: 1 h daily for 4 weeks	-MDA (−) -SOD (+) -Apoptosis (−)	10 mmol/kg/day, i.p., for 4 weeks before the exposure to cigarette smoke	Rabbits	[49]
Ethanol: 3 mg/kg, oral	-TOS (−) -TAS (=) -Histopathological assessment	10 μmol/kg, i.p., immediately after ehanol administration	Rats	[50]
Acrolein: 1 M	-Cell viability (+) -ROS (−) -GSH (+) -MAPKs -Akt/GSK3 -α/β-secretase	0-90 μM, pretreatment for 30 min	HT22 mouse hippocampal cells	[51]
K2CrO4: 2 mg/kg/day, i.p., for 30 days	-SOCS3, JAK2 and STAT3 -NO (−) -GSH (+) -SOD (+) -GSH-Px -AChE -TNF-α (−) -IL-6 (−)	20 mg/kg/day, orally, for 30 days	Wistar male rats	[52]
CdCl2: 1.5 mg/kg	-Neurobehavioural assessment -Histopathological assessment -AMPK and pAMPK -SIRT1 -Bcl-2 (+) -Bax (−) -Caspase-3 (−) -p-Tau -TLR4 -IL-6 (−) -IL1-β (−) -TNF-α (−)	10 μmol/kg/ day, for 4 weeks	7 weeks old Kunming mice	[53]
Chlorpyriphos: 10 mg/kg, oral	-AChE (−) -TOS (−) -TAR -Histopathological assessment -Caspase-3 -Bcl-2 -Bax	10 μmol/kg, i.p., immediately after chlorpyriphos admnistration	Wistar rats	[54]
INH and ETM: 50 mg/kg/day, orally, for 30 days	-Histopathological assessment -MDA (−) -TOS (−) -TAC (+) -SOD (+) -PON-1 (+) -NO (−)	10 mol/kg/day, i.p., for 30 days	Male Sprague-Dawley rats	[55]
Sevoflurane: (2.9%) for 6 h at day 7	-Caspase-3, -8 and -9 (−) -Bax (−) -Bcl-2 (+) -Bcl-xL (+) -Bad (−) -MAPK (−) -JNK -ERK -PI3K (−)	10, 20 or 40 mg/kg, from postnatal day 1 to day15	Rat pups	[56]

**Table 3:** CAPE protective effects against ischemia/reperfusion injury. Where applicable, the effect of CAPE on the parameters measured is reported between parentheses (+ or −).

Stimulus	Parameters Measured	CAPE Dose	Animal Used	Ref.
Bilateral CCA occlusion (20 min) then reperfusion (20 min)	-ADA -XO -SOD -GSH-Px -CAT -NO -MDA (-)	10 µmol/kg, i.p., 10 min. after placing the occlusive vascular clamps	Sprague–Dawley rats	[57]
Cerebral infarction: right MCA occlusion and bilateral CCA clipping, 60 min	-NO (+) -Histopathological assessment	0.01, 0.1, 1 and 10 µg/kg, i.v. 15 min before MCA occlusion	Male Long–Evans rats	[58]
MCA occlusion, 20 or 90 min	-Histopathological assessment -Neurological assessment -TNF-α (−) -IL-1β (−) -iNOS (−) -ED1 (−) -Bcl-xL (+) -Caspase-3 (−) -NO (+) -TBARS -GSH (+) -NF-κB (−) -MDA (−) -ICAM-1 (−) -E-selectin (−) -Nitrotyrosine (−)	1–10 mg/kg, i.v., either at or after reperfusion	Male Sprague–Dawley rats	[59]
Right permanent MCA occlusion	-Histopathological assessment -Neurological assessment -MDA (−) -GSH (+) -CAT (−) -NO (+) -XO (−)	10 µmol/kg/day, i.p., after occlusion for 7 days	Male New Zealand rabbits	[60]
Permanent MCA occlusion	-Serum S-100B (−)	10 µg/kg/day, i.p., for 7 days after occlusion	Male New Zealand rabbits	[61]
MCA occlusion (60 min), followed by 24 h reperfusion	-Structural changes	50 µM/kg, i.p., once before occlusion	Wistar rats	[62]
Cortical ischaemia: skull irradiation with cold light laser in combination with systemic administration of rose bengal	-Histopathological assessment -TNF-α (−) -HIF-1α (−) -MCP-1 (−) -IDO (−) -HO-1(+) -IL-1α (−) -IL-10 (+)	0.5–5 mg/kg, i.p., 1 and 6 h after ischaemic insult	Male C57BL/6 mice	[63]
Anoxia-reoxygenation	-Mitochondrial oxygen consumption -Mitochondrial anisotropy -Mitochondrial TBARS -Mitochondrial protein concentrations -Protein carbonylation (−) -CL and Cytochrome c release (−)	10–10^−5^ μM before the anoxia or just at reoxygenation	Male Kunming mice	[64]
CCA ligation followed by exposure to hypoxia	-i- and nNOS (−) -Cytochrome c (−) -Caspase-1 and -3 (−)	40 mg/kg/day, 4 hrs before and/or after the stimulus	7-day-old Sprague–Dawley rats	[65]
SAH	-MDA (−) -GSH (+) -NO (+) -Histopathological assessment	10 µmol/kg, i.p., twice daily for 5 days after SAH	15-week-old male Wistar rats	[66]
SAH	-Histopathological assessment	10 mg/kg/day, twice a day, for 3 days starting 6 h after SAH.	Wistar rats	[67]
Aortic occlusion, 21 min	-MDA (−) -SOD -CAT -Histopathological assessment -Neurologic assessment	10 µmol/kg, i.p. 30 min before the stimulus	New Zealand rabbits	[68]

**Table 4:** Protective effects of CAPE against injury. Where applicable, the effect of CAPE on the parameters measured is reported between parentheses (+, − or =).

Stimulus/Injury	Parameters Measured	CAPE Dose	Animal/Cell Used	Ref.
SCI: aneurysm clip	-IL-1β (−) -TNF-α (−) -Histopathological parameters	10 μg/kg, i.p., 30 min after trauma	Male Wistar rats	[69]
Paraplegia: epidural clip application for 60 s.	Histopathological parameters	10 μmol/kg, i.p.	Female Sprague-Dawley rats	[70]
SCI: Yasargil aneurysm clips	-IL-6 (−) -TNF-α (=) -Histopathological parameters	1 μg/kg, following SCI induction	Female Wistar rats	[71]
SCI: Yasargil aneurysm	-MDA (−) -TOA (−) -TAC (+) -SOD (−) -GSH-Px	1 μg/kg, single dose	Female Wistar rats	[72]
SCI: hemitransection	-Locomotor function -Histopathological parameters -IL-1β (−) -iNOS (−) -COX-2 (−)	2 or 10 μmol/kg/day, i.p., for 28 days	Female Wistar rats	[73]
Head trauma with marmarou model	MPO activity (−)	10 mg/kg, i.p., 24 h before trauma and 30 min after trauma and every day for 7 days	Male Sprague mice	[74]
Brain trauma: CCI injury model	-Blood–brain barrier (BBB) integrity (+) -Claudin-5 expression (+) -Neurobehavioural assessment	10 mg/kg, i.p., -30 min following injury and/or daily for the next 4 days	-Male Sprague-Dawley rats -C57BL/6 mice	[75]
TBI: using cranial impact to the skull from a height of 7 cm at a point just in front of the coronal suture and over the right hemisphere.	-MDA (−) -SOD (+) -GSH-Px (+) -CAT (=) -Histological examinations -Caspase- 3	10 μmol /kg/i.p., single dose15 min after trauma	Male Sprague–Dawley rats	[76]
TBI: focal CCI technique	-Neurobehavioural parameters -Histopathological parameters -AMPK and pAMPK (+) -Fission (Drp1 and Fis 1) and fusion (Opa1)-associated proteins (+) -Mitochondrial factor PGC1α -HO-1 -MnSOD (+)	5 mg/kg, plus GSNO 0.05 mg/kg, 2 h after CCI, i.v. and then daily orally	Young adult male wild type C57BL/6 mice	[77]

**Table 5:** CAPE anti-tumoral effects in CNS. Where applicable, the effect of CAPE on the parameters measured is reported between parentheses (+ or −).

Stimulus/Tumor	Parameters Measured	CAPE Dose	Ref.
Human astrocytoma (CRT-MG cells)	-Cell viability (−) -ROS (−) -Caspase-3 and -8 (+) -NOX4 -DEVDase activity	0–25 µg/mL pre-treatment for 1 h	[79]
Malignant brain tumor: human U87MG glioma cells	-PLD (−) -PLD1 (−) -PLD2 -NF-κB-binding motif (−) -α-tubulin -MMP-2 (−) -Invasion assay (−) -Gelatin zymography	10, 20 µM for 24 h	[80]
-HEK293 cells stably co-transfected with a pcDNA3.1 vector expressing 5-LO and a pBUDCE4.1 vector expressing 5-LO activating protein -Human glioma cells: Hs683 and LN319.	-Cell viability (−) -Molecular Docking -5-LO activity (−)	-1 μM, pre-incubation -10 µM, for 3 days	[81]
Tumor xenografts in nu/nu mice: SC injection of *NF1*-deficient MPNST (S-462) cells or *NF2*-deficient Schwannoma (HEI-193) cells	-Cell viability (−) -Tumor size (−)	Bio 30 (a CAPE-rich extract), 100–300 mg/kg, i.p., twice a week	[82]

## Abbreviations

5-LO	5-lipoxygenase
6-OHDA	6-Hydroxydopamine
AChE	acetylcholinesterase
AD	Alzheimer’s disease
ADA	adenosine deaminase
AIF	Apoptosis-inducing factor
ALS	Amyotrophic lateral sclerosis
ALT	alanine transaminase
AST	aspartate transaminase
AMN	adrenomyeloneuropathy
AMPK	5′-adenosine monophosphate-activated protein kinase
Bax	Bcl-2-associated X protein
Bad	Bcl-2-associated agonist of cell death
BBB	blood brain barrier
Bcl-2	B cell CCL/lymphoma 2
Bcl-xL	Bcl-2-like 1
BDNF	brain-derived neurotrophic factor
CAPE	caffeic acid phenethyl ester
CAT	Catalase
CCA	common carotid arteries
CCI	controlled cortical impact
CCL- 2	C-C.motif ligand-2
CGNs	cerebellar granule neurons
CNS	central nervous system
COMT	catechol *O*-methyltransferase
COX-2	cyclooxygenase-2
CPF	Clorpyrifos-ethyl
CREB	cAMP-responsive element binding protein
DA	dopamine
DCF	dichlorofluorescein assayDHHC: dihydroxydihydrocinnamic acid phenethylester
DMC	dimethoxycinnamic acid phenethyl ester
EAE	experimental autoimmune encephalomyelitis
ED1	marker of activated macrophage/microglia
eNOS	endothelial nitric oxide synthase
ERK	extracellular signal-regulated kinase
EPO	erythropoietin
ETM	ethambutol
FAPE	ferulic acid esterGSH: glutathione
GSH-Px	glutathione peroxidase
GSNO	S-nitrosoglutathione
HE	encephalopathy
HIF-1α	hypoxia inducing factor-1α
HO-1	heme oxygenase
ICAM-1	Intercellular adhesion molecule-1
ICV	intracerebroventricular
IDO	indoleamine 2,3-dioxegenase
IFN- γ	interferon-γ
IFOS	ifosfamide
IκB	inhibitor κB
IKK	inhibitor of nuclear factor-κB (IκB) kinase
IL-1β	interleukine-1β
INH	isoniazid
iNOS	inducible nitric oxide synthase
JNK	c-Jun N-terminal kinase
LPS	lipopolysaccharide
MAO	monoamine oxidase
MAPK	mitogen-activated protein kinases
MBP	Myelin Basic Protein
MCA	middle cerebral artery
MCP-1	monocyte chemoattractant protein-1
MDA	Malondialdehyde
MK-801	dizocilipine maleate
MKK4	Mitogen-Activated Protein Kinase Kinase 4
MMP	matrix metalloproteinase
MP	Methylprednisolone
MPO	myeloperoxidase
MPP+	1-methyl-4-phenylpyridinium
MPTP	methyl-4-phenyl-1,2,3,6-tetrahydropyridine
MS	multiple sclerosis
MTX	methotrexate
NF	neurofibromatosis
NF-κB	nuclear factor-κB
NGF	nerve growth factor
NMDA	N-methyl-D-aspartate
NO	nitric oxide
Nrf2	nuclear factor erythroid 2-related factor 2
PAMPA	parallel artificial membrane permeability assay (PAMPA)PC: protein carbonyl
PD	Parkinson’s disease
PFC	prefrontal cortex
PI3K	phosphoinositide 3-kinase
PLD1	phospholipase D1
PMNL	polymorphonuclear leukocytes
PON1	paraoxonase
PTZ	pentylenetetrazole
RMN	rostral mesencephalic neurons
ROS	reactive oxygen species
SAH	Subarachnoid hemorrhage
SCI	spinal cord injury
SE	status epilepticus
SOD	superoxide dismutase
STZ	streptozotocin
TAC	total antioxidant capacity
TAK1	transforming growth factor-β-activated kinase 1
TAR	total antioxidant response
TAS	total antioxidant status
TBARS	thiobarbituric acid reactive substances
TBI	traumatic brain injury
TH	tyrosine hydroxylase
TNF-α	tumor necrosis factor-α
TOA	total oxidant activity
TOC	total oxidant capacity
TOS	Total oxidant status
TRAF2	TNF Receptor Associated Factor 2
TSA	total sialic acid
VLCFA	very long chain fatty acids
X-ALD	X-linked adrenoleukodystrophy
XO	xanthine oxidase
